# The relative effects of parental alcohol use disorder and maltreatment on offspring alcohol use: Unique pathways of risk

**DOI:** 10.1017/S0954579423001347

**Published:** 2023-10-31

**Authors:** Andrew J. Ross, Justin Russotti, Sheree L. Toth, Dante Cicchetti, Elizabeth D. Handley

**Affiliations:** 1 Mt. Hope Family Center, University of Rochester, Rochester, NY, USA; 2 Institute of Child Development, University of Minnesota, Minneapolis, MN, USA

**Keywords:** Alcohol dependence, maltreatment, peers, symptomatology

## Abstract

Childhood adversity represents a robust risk factor for the development of harmful substance use. Although a range of empirical studies have examined the consequences of multiple forms of adversity (i.e., childhood maltreatment, parental alcohol use disorder [AUD]), there is a dearth of information on the relative effects of each form of adversity when considered simultaneously. The current study utilizes structural equation modeling to investigate three unique and amplifying pathways from parental AUD and maltreatment exposure to offspring alcohol use as emerging adults: (1) childhood externalizing symptomatology, (2) internalizing symptomatology, and (3) affiliation with substance-using peers and siblings. Participants (*N* = 422) were drawn from a longitudinal follow-up study of emerging adults who participated in a research summer camp program as children. Wave 1 of the study included 674 school-aged children with and without maltreatment histories. Results indicated that chronic maltreatment, over and above the effect of parent AUD, was uniquely associated with greater childhood conduct problems and depressive symptomatology. Mother alcohol dependence was uniquely associated with greater affiliation with substance-using peers and siblings, which in turn predicted greater alcohol use as emerging adults. Results support peer and sibling affiliation as a key mechanism in the intergenerational transmission of substance use between mothers and offspring.

## Introduction

Childhood adversity represents a robust risk factor for the development of harmful substance use (Hughes et al., 2017; Dube et al., 2003) and substance use disorders (SUDs; Brown & Shillington, 2017), with over 25% of all cases of SUD attributed to experiencing early adversity (Kessler et al., 2010). Early adversity can include, but is not limited to, exposure to abuse and neglect and living with a caregiver who is unable to adequately meet their needs due to substance misuse (Chassin & Handley, 2006; Smith, Johnson, Pears, Fisher, & DeGarmo, 2007), domestic violence (Renner & Slack, 2006), or mental health challenges (Park et al., 2006). Moreover, different forms of early adversity tend to co-occur within families (e.g., Brown & Shillington, 2017; Brown et al., 2019; Russotti et al., 2021) which may increase the likelihood of negative consequences for offspring, including substance use (Brown & Shillington, 2017; Fettes et al., 2013) and delinquency (Grogan-Kaylor et al., 2008; Ryan & Testa, 2005).

Given the clinical and public health implications for experiencing any form of childhood adversity, the current study examines the effects of two forms of early adversity in particular (i.e., living with a parent who abuses substances; exposure to maltreatment) given their highly co-occurring nature (e.g., Appleyard et al., 2011; Hayre et al., 2019). Children whose parents abuse drugs and alcohol are almost three times more likely to experience parental neglect relative to children of parents who do not abuse substances (Smith & Wilson, 2016). Being raised by a parent with a SUD has been identified as one of the most common reasons children enter the child welfare system (Olsen, 2015; Seay, 2015; Straussner & Fewell, 2015). National child welfare system data indicate that substance abuse is identified or suspected in 66% of all substantiated cases within the child protective service system and in 79% of cases that involve removal of children from their home (U.S. Department of Health and Human Services, 2021).

Although a range of empirical studies have examined the consequences of these forms of adversity occurring together, there is a dearth of information on the relative effects of each form of adversity when considered simultaneously. It appears that only one prior study, conducted by Stein et al. (2002), examined the relative contributions of parent substance use and childhood maltreatment. Specifically, they found that among homeless adult women, exposure to parent substance use directly predicted the development of later substance use problems, while childhood abuse (i.e., physical abuse, sexual abuse, emotional abuse), not assessing for neglect, indirectly predicted substance use problems via self-esteem and later abuse pathways. From the perspective of clinical prevention and intervention development, it is essential to parse out the specific influences of different adverse experiences as therapists and policymakers develop targets for therapeutic improvement.

Moreover, the current study examines pathways through which these adverse familial experiences confer risk for alcohol misuse in offspring during emerging adulthood, given the importance of this developmental period in the context of substance use. The use of alcohol and drugs often follows a developmental pattern, as substance use most commonly begins in adolescence, escalates in young adulthood, and peaks between the ages of 18 and 25 years (Hussong & Smith, 2018). Among the processes that can set in motion substance use throughout adolescence and emerging adulthood, parent substance use (e.g., Chassin et al., 2019; Knight et al., 2014), and maltreatment (e.g., Cicchetti & Handley, 2019; Jonson-Reid et al., 2012) have each been identified as robust predictors.

### Intergenerational transmission of substance use

Offspring of substance-abusing parents are among the highest risk groups for experiencing alcohol use disorders (AUDs) and other SUDs (Bountress et al., 2017; Chassin et al., 2016; Hussong et al., 2011). A summary of the National Surveys on Drug Use and Health in the United States from 2009 to 2014 found that about 8.7 million or 12.3% of American children aged 17 or younger lived in households with at least one parent who had a past-year SUD (Straussner & Fewell, 2018). Extant research highlights the potential for multifinality in the developmental trajectories of youth who have a parent with a SUD, as some offspring of parents with alcohol and drug use disorders can demonstrate resilience throughout their youth and into adulthood, while many offspring of adults with SUD can develop a range of emotional, behavioral, physical, cognitive, and social problems (Cicchetti & Rogosch, 1999; Straussner & Fewell, 2015).

Capaldi et al. (2016) found that maternal alcohol use was associated with their child’s earlier onset and use of alcohol, while those who engage in heavy drinking during adolescence likely continue this problematic drinking into emerging adulthood (Diggs & Neppl, 2018; Thompson et al., 2014). Specifically, Diggs and Neppl (2018) examined the continuity of alcohol use from middle adolescence to emerging adulthood, finding that alcohol use at age 16 predicted binge drinking in late adolescence, which led to binge drinking in emerging adulthood. Additionally, Henry and Augustyn (2017) found that individuals who began using cannabis by age 15 were more likely to meet criteria for a lifetime cannabis disorder. As such, there is support for a cascading effect of parents’ substance use on the substance use of their offspring when they reach young adulthood. Taken together, continued examination of the pathways of risk and resilience for these offspring can provide greater insight into the implementation of developmentally attuned preventive interventions (Hussong et al., 2011; Ialongo et al., 2006).

In conjunction with parenting factors that may influence adolescent and young adult substance use, there is evidence that additional family- and community-level characteristics play an important role in the development of substance use during these important developmental periods (e.g., Heerde et al., 2019). Children of mothers with SUDs are more often exposed to violence and crime in and outside of the home, which can contribute to cumulative risk for future developmental problems (Hser et al., 2015). Families affected by SUDs are more likely than other families to experience a variety of stressful life events, including violence, physical health challenges, economic hardship, and family disruption (Hoffmann et al., 2000; Hoffmann & Cerbone, 2002). These family- and community-level stressors can in turn increase the likelihood of adolescent stress and ultimately heighten risk for adolescent and young adult drug use and abuse, as these individuals try to cope with the hardships endured (Hoffmann & Cerbone, 2002).

### Effects of maltreatment on substance use

Child maltreatment represents an early experience that is among the most detrimental to children’s psychological, social, and biological development (Cicchetti & Toth, 2016) and is essential to consider in the context of both parental substance use and the development of adolescent and young adult substance use. Individuals who are raised by one or more parents with SUD are at substantially greater risk for exposure to child maltreatment (e.g., Appleyard et al., 2011; Cicchetti & Handley, 2019). Exposure to maltreatment is predictive of delinquency and substance use among adolescents (e.g., Cicchetti & Handley, 2019; Jonson-Reid et al., 2012) and young adults (e.g., Capusan et al., 2021), including an earlier initiation into drinking (Dube et al., 2006) and persistently elevated heavy episodic drinking throughout adolescence and young adulthood (Shin et al., 2013). Prior empirical work has identified child maltreatment as a unique predictor of persistent alcohol dependence in adulthood over and above other forms of childhood adversity (e.g., parental divorce, parental death; Elliot et al., 2014).

Individuals whose formative years are marked by trauma and adversity within the family system, notably maltreatment (e.g., abuse, neglect), are more likely than others to experience accelerated biological and social maturation and “rush to adulthood” (Belsky et al., 2015; Ryan et al., 2015). Youth with histories of maltreatment may seek out early transitions to adulthood in order to escape a stressful or traumatic environment. Prior research suggests that maltreatment victimization is associated with an increased likelihood of dropping out of high school (e.g., Allwood & Widom, 2013), prematurely leaving the family home to live on one’s own (e.g., Thrane et al., 2006; Tyler & Bersani, 2008), and adolescent pregnancy (e.g., Russotti et al., 2022). In turn, the heightened access to and desire for drugs and alcohol may be a consequence of, or may co-occur with, this accelerated transition into adulthood (Augustyn et al., 2019).

### Pathways to problem drinking

#### Externalizing pathway

Ongoing research efforts have provided the field with a growing understanding of the potentially overlapping pathways through which parent substance use and maltreatment can influence offspring development and behavior. There is robust support for an externalizing pathway to offspring substance use stemming from parental substance use (e.g., Chassin et al., 2013, 2016) and maltreatment (e.g., Handley et al., 2015; Oshri et al., 2011). This pathway, also referred to as the behavioral disinhibition pathway, is marked by childhood behavioral concerns including aggression, poor self-regulation, and rule-breaking behaviors, which are associated with increased substance use in adolescence and beyond (Chassin et al., 2016; Cicchetti & Handley, 2019).

Parents may deter their adolescents’ substance use via parenting behaviors (e.g., social support, socialization about substance use; Cicchetti & Handley, 2019; Handley & Chassin, 2013). However, in families consisting of a parent with a SUD or in families affected by maltreatment, these substance-deterring parenting behaviors can be compromised (e.g., Macaulay et al., 2005; Wade & Pevalin, 2005; Wall & Kohl, 2007). Furthermore, maladaptive parenting practices and home environments often interact with offspring behavior and affiliation with substance-using peers to enhance risk (Chassin et al., 2013; Cicchetti & Handley, 2019).

#### Internalizing pathway

A related but separate pathway underlying risk for the development of substance use is the internalizing pathway. This pathway to SUD, also referred to as the negative affect pathway, is marked by an early inhibited temperament style and internalizing symptoms throughout childhood continuing into adolescence and adulthood (Cicchetti & Handley, 2019; Hussong et al., 2011). Within the internalizing framework, individuals exposed to early adversity (e.g., abuse) may exhibit prolonged substance use during adolescence and adulthood as a means of escape or self-medication (Hussong et al., 2011; Lansford et al., 2010). As noted by Cicchetti and Handley (2019), empirical support for the internalizing pathway to the development of SUD has been mixed. Although some prior studies have demonstrated that internalizing symptoms predict problematic drinking behaviors above and beyond the effects of externalizing symptoms (e.g., Menary et al., 2017; Pesola et al., 2015) others have observed a lack of an association (e.g., Chassin et al., 1999; King & Chassin, 2008) or a protective effect of internalizing symptoms, such that social withdrawal reduced risk for engagement in substance use (e.g., Kaplow et al., 2001; Rogosch et al., 2010). Within the context of child maltreatment, evidence for an internalizing pathway is emerging (e.g., Handley et al., 2017; Jester et al., 2015; Mezquita et al., 2014), such that childhood abuse is associated with higher levels of negative emotionality, in turn predicting motivation to engage in substance use. Moreover, there is growing support for an internalizing pathway from parent SUD to offspring substance use (e.g., Chassin et al., 2016; Hussong et al., 2011).

#### Peer affiliation pathway

In addition to the symptomatology pathways, there is a range of extant empirical work underscoring the role of peer relationships and affiliation with deviant peers in the development of risk behavior among individuals reared in high-risk households (e.g., Chen et al., 2005; Salzinger et al., 2007). Exposure to maltreatment has been consistently identified as a predictor of maladaptive peer relationships (e.g., Bolger & Patterson, 2001; Kim & Cicchetti, 2010). Youth with maltreatment histories tend to have poor quality, unsatisfactory friendships marked by aggression and coercion (Cicchetti & Toth, 2016; Dodge et al., 1994). Affiliation with substance-using peers may be driven by a motivation for belongingness; however, peer pressure, control, manipulation, and social reinforcement among peer groups engaging in substance use may be particularly pronounced among individuals with histories of early adversity (Ellis & Wolfe, 2009).

Affiliation with deviant, substance-using peers has been identified as one of the most robust predictors of substance use throughout adolescence and into emerging adulthood (e.g., Chassin et al., 2013; Chuang et al., 2005; Haller et al., 2010). Moreover, with respect to being reared by a parent experiencing SUD, affiliation with substance-using peers has been shown to mediate the effects of parenting behaviors on youth substance use (Kiesner et al., 2010). Youth experimentation with alcohol may be viewed as modeling peer or older sibling behavior. Social learning theory suggests that peer or sibling substance use may have a modeling effect (Bandura, 1977), by which substance use behaviors of peers and siblings encourage imitation by adolescents. Further, being raised in a household in which alcohol use is occurring frequently, used as a coping strategy by parents and older siblings, and viewed as normative and appropriate may encourage youth to seek out peers who share similar positive attitudes about drinking. Parental substance use has also been demonstrated to decrease the amount of monitoring parents provide to adolescents (e.g., Chassin et al., 1996; Sher et al., 2005) with fewer restrictions imposed regarding engagement with peers. This decreased monitoring can in turn promote greater accessibility of substances and heavier use of substances among adolescents (Clark et al., 2012; Van der Vorst et al., 2006). In sum, exposure to early adversity in the form of maltreatment and parental AUD may confer risk for problematic alcohol use in emerging adulthood as a result of three not mutually exclusive pathways: externalizing symptoms in childhood, internalizing symptoms in childhood, and affiliation with a substance-using peer network.

### Current study

Although parental alcohol use and maltreatment exposure have been separately identified as predictors of offspring alcohol use and established as highly co-occurring (e.g., Appleyard et al., 2011), there is limited information on the specific contributions of parental alcohol relative to maltreatment when considered in tandem. Some extant empirical work suggests a cumulative nature to adverse childhood experiences (ACEs) in their effects on youth and adult health, such that the summation of adversity (e.g., parental substance abuse, maltreatment exposure) would have greater effects on the development of offspring substance use during young adulthood, in addition to various aspects of well-being and functioning (Hughes et al., 2017; Rogers et al., 2022). However, this prior work does not appear to parse out the specific unique contributions of each familial exposure, nor does it consider how maltreatment exposure and parent alcohol use disorder may interact, or amplify, each other. Specifically, the interaction of maltreatment and parental AUD may be better represented by a synergistic amplification model, such that the effects of maltreatment on an individual may be exacerbated when the individual is additionally reared in a household affected by parent AUD. Although prior literature provides support for the process of amplification among co-occurring forms of adversity (e.g., being reared in a household with parental psychopathology; Carter et al., 2001; Sellers et al., 2013), this remains unclear in the context of co-occurring maltreatment and parent alcohol dependence.

In the current study, we investigate the potential unique and amplifying pathways from parental AUD and maltreatment exposure to offspring development of concerning alcohol use. Our first aim is to examine the unique effects of maltreatment, parental AUD, and their interaction, on offspring development of problematic alcohol use in emerging adulthood. We hypothesize that parental AUD and maltreatment would have an amplifying effect, such that relative to those only affected by one of these adverse experiences, the influence of maltreatment on the development of problematic alcohol use in young adulthood would be stronger among individuals additionally exposed to parental AUD. Our second aim is to examine the role of multiple, and not mutually exclusive, theoretically and empirically supported pathways from parental AUD and maltreatment to offspring alcohol use, specifically the development of problem alcohol use via 1) childhood externalizing symptomatology, 2) childhood internalizing symptomatology, and 3) affiliation with substance-using peers and siblings during childhood. We hypothesize that greater externalizing symptomatology, greater internalizing symptomatology, and affiliation with peers and siblings engaging in substance use would differentially mediate the effects of experiencing both parental AUD and maltreatment.

## Method

### Participants

Participants (*N* = 422) were drawn from a longitudinal follow-up study of emerging adults who participated in a research summer camp program as children. The original study (wave 1) included 674 low-income maltreated (*n* = 348) and non-maltreated (*n* = 326) children aged 10 to 12. The original sample was racially and ethnically diverse (71.6% Black, 11.8% White, 12.6% Latinx, 4.0% biracial, 1% other race) and evenly distributed by gender (50.1% male). Most children were from single-parent families (68.7%) with a history of receiving public assistance (96.1%). With respect to parental characteristics, 41.6% of participants were reared by a mother with at least a high school degree or GED equivalent. At wave 2 (W2), emerging adults were on average 19.68 years old (SD = 1.15), 51.1% female, 53.5% (*n* = 207) exposed to maltreatment, and identified as Black (68.5%), White (9.2%), Latinx (12.6%), biracial (6.1%), and other race (3.6%). W2 participants were demographically comparable to those at W1 on sex (*χ*^2^ (1) = 1.98, *p* = .34) and race (*t* (674) = 2.31, *p* = .28). Additionally, W2 participants did not significantly differ from the W1 participants lost to attrition on maltreatment status (*χ*^2^ (1) = .41, *p* = .52), income (*t* (674) = 1.20, *p* = .58), sex (*χ*^2^ (1) = 1.58, *p* = .21), childhood conduct problems *t* (674) = 0.43, *p* = 0.67) or internalizing symptoms (*t* (674) = 1.26, *p* = 0.21).

### Procedure

W1 participants were recruited for a summer research camp from 2004 to 2007. Children in the maltreated group had substantiated investigations of child maltreatment according to Department of Human Services (DHS) Child Protective Services (CPS) records. Children without CPS involvement were recruited from families receiving Temporary Assistance to Needy Families (TANF) to ascertain a sociodemographically comparable sample of children without maltreatment experiences. A DHS liaison identified eligible families and contacted a random sample from both groups via mail. If families elected to participate, their contact information was shared with research staff. Parents who chose to enroll their children in the research summer camp provided signed consent to study procedures. Children also provided assent to study procedures.

During the camp, counselors facilitated recreational activities (e.g., music, art, sports) with the same groups of 6–8 children (35 hours of direct contact and observation). Children were assigned to same-gender peer groups for participation in recreational activities in order to reduce the risk of moderation by gender and to reflect the naturally occurring interpersonal processes among school-aged children. Half of the children in each of the groups were maltreated and the other half did not have a history of maltreatment. Maltreatment status was unknown to camp counselors, who were trained on completing assessments based on observations and interactions with the children in their respective groups. Children self-reported on their functioning and camp counselors provided independent ratings of childhood functioning after the end of the week. At W2 (~eight years after W1), a variety of strategies were used to relocate and recruit W1 participants for a follow-up study during emerging adulthood. Records of last known addresses, extensive public internet searches (e.g., LexisNexis), contact information from medical records, and neighborhood canvasing were part of a comprehensive recruitment design. Interested participants completed signed consents and then participated in three research visits.

### W1 measures

#### Child maltreatment

Specific maltreatment experiences were coded from DHS records using the Maltreatment Classification System (MCS; Barnett et al., 1993) by trained doctoral students and by clinical and developmental psychologists. The MCS is a reliable and valid method for classifying maltreatment (Manly, 2005) that utilizes DHS records detailing investigations and findings involving maltreatment in identified families over time. Rather than relying on official designations and case dispositions, the MCS codes all available information from DHS records, making independent determinations of maltreatment experiences. Based on operational criteria, the MCS designates subtypes of maltreatment children have experienced (i.e., neglect, emotional maltreatment, physical abuse, and sexual abuse) and the severity of each type on a scale ranging from 1 (least) to 5 (most) severe. Events were coded as occurring during five developmental periods, including infancy (0–12 months), toddlerhood (13–36 months), preschool (36–60 months), early school age (age 5–7), and later school age (age 8–12), with number of developmental periods utilized as a predictor in our analytic model. Among participants exposed to maltreatment, 56.6% experienced maltreatment during one developmental period, 26.4% during two developmental periods, 11.2% during three periods, 4% during four periods, and 1.7% during five developmental periods. Coders were required to meet acceptable reliability with criterion standards before coding actual records for the study; weighted κ’s with the criterion ranged from .86 to .98. Reliabilities (κ’s) for the presence vs. absence of maltreatment subtypes ranged from .90 to 1.00.

#### Mother alcohol dependence

The Diagnostic Interview Schedule for DSM-IV (DIS-IV; Robins et al., 1995) is a structured clinical interview administered in person with the help of a computer system to provide clinical psychiatric diagnoses and symptom counts based on DSM-IV criteria (American Psychiatric Association, 1994). Modules of the DIS-IV were conducted with caregivers in a private room by administrators trained on this measure. For the current study, we examined lifetime alcohol dependence symptoms among mothers, ranging from 0 to 7 symptoms.

#### Childhood conduct problems

Childhood conduct problems were assessed using the Pittsburgh Youth Survey (PYS; Loeber et al., 1998). The PYS is a self-report measure that examines a range of delinquent behaviors and substance use in childhood. Reporters indicate both the lifetime prevalence of behaviors, as well as the occurrence in the past six months. A total of the 25 conduct disorder symptoms (e.g., stealing, cheating on school tests, damaging property) endorsed in the past 6 months, excluding the 6 substance use items, was used to determine childhood conduct problems. The mean number of symptoms endorsed was 2.21 (*SD* = 2.80). Reliability was adequate for the 25 items (α = .83).

#### Childhood depressive symptoms

Child depressive symptoms were measured using child self-report of the Child Depression Inventory (CDI; Kovacs, 1982). The CDI is 27-item questionnaire measure of depressive symptoms designed for school-age children. Children were asked to select the response option that best describes them within the past two weeks. The CDI is a well-established measure of child depressive symptoms with good psychometric properties (Kovacs, 1982). A summary score of the 27 items was calculated to index self-reported depressive symptoms. Participants in this study reported a mean of 8.01 (*SD* = 6.76). A total score greater than, or equal to 19 is indicative of clinical level depressive symptoms (Kovacs, 1982). In the current sample, 8.1% of children met this threshold for clinically elevated depressive symptoms.

#### Affiliation with substance-using peers and siblings

Peer and older sibling substance use were measured separately using two 7-item self-report questionnaires. Children reported how many of their friends use cigarettes, alcohol, marijuana, pills, cocaine, paint, glue, or other substances, to their knowledge using a 6-point Likert-type scale (i.e., 0 = none, 1 = a few, 2 = some, 3 = most, 4 = all, 5 = don’t know). They separately reported if they have an older brother or sister, and if so, whether their sibling uses any of the listed substances with “yes,” “no,” and “don’t know” responses. For the purposes of the current study, and in consideration of the developmental level of participants and their peers, we created a sum score of only the three items assessing cigarette, alcohol, and marijuana use for peers, and separately for older siblings, which were then summed to create a composite peer and older sibling substance use score. This composite was then converted into a binary variable, such that the absence of substance use among peers or siblings was coded as 0 and a sum of 1 or above (i.e., presence of any substance use) was coded as 1. Among participants, 22.3% reported peer use of at least one substance and 18.2% reported sibling use of at least one substance; 33.5% reported peer and/or sibling use based on the composite score created. Participants were assured that their responses would be confidential, and this information would not be used to get them or their friends and siblings into trouble.

### W2 measures

#### Offspring emerging adult problem drinking

##### Emerging adult alcohol dependence.

The Diagnostic Interview Schedule for DSM-IV (DIS-IV; Robins et al., 1995) was administered with offspring at the W2 measurement period to identify clinical psychiatric diagnoses and symptom counts based on DSM-IV criteria (American Psychiatric Association, 1994). For this wave of the study, modules of the DIS-IV were conducted with emerging adult offspring in a private room by administrators trained on this measure. For the current study, we examined past-year symptoms of alcohol dependence among young adults, ranging from 0 to 7 symptoms.

##### Emerging adult alcohol consumption.

Problem drinking in emerging adulthood was assessed using 4 alcohol consumption items, which were adapted from Sher (1993)) and subsequently utilized in a plethora of empirical work addressing the developmental consequences of youth and adult alcohol use (e.g., Chassin et al., 1999, 2013). Two items asked participants to self-report their quantity of hard liquor consumption, ranging from (0) “No drinks” to (8) “Nine or more drinks” during a given drinking session, and separately, frequency of consumption of hard liquor in the past year, with response options ranging from (0) “Never” to (7) “Everyday.” These items were multiplied to index the consumption of hard liquor in the past year. Because this consumption variable was nonnormality distributed, and given that modeling techniques are sensitive to nonnormality, a square root transformation was used to reduce skewness and kurtosis. Participants also self-reported the frequency of their binge drinking of beer, wine, wine coolers, or hard liquor within the past year (defined as 3 or more drinks for women and 5 or more drinks for men) and the frequency of getting drunk on alcohol (not just lightheaded) within the past year. The response scale for frequency of binge drinking and drunkenness ranged from (0) “Never to (7) “Everyday.” As described in detail below, these three variables (i.e., consumption of hard liquor, binge drinking, and drunkenness), in addition to a diagnosis of alcohol dependence from the DIS-IV, were modeled as indicators of an offspring emerging adult problem drinking latent construct in subsequent analyses. In this sample, 70.5% of emerging adult participants indicated drinking alcohol in the past year.

### Data analytic plan

Zero-order correlations, descriptive data analyses, confirmatory factor analysis (CFA), and structural equation models (SEM) were estimated using Mplus Version 8.0 (Muthén & Muthén, 2017). CFA was estimated to examine model fit of an endogenous latent factor representing offspring problematic alcohol use at emerging adulthood. The CFA was specified such that past-year consumption of hard liquor, binge drinking, drunkenness, and alcohol dependence symptoms based on the DIS-IV, were indicators of a latent factor representing offspring problematic alcohol use (see above for measurement details of the four indicators). Results of this measurement model informed specification of the structural model.

The structural equation model was first specified such that the number of developmental periods in which individuals experienced maltreatment, and the number of mother alcohol dependence symptoms, were included as exogenous predictors, as well as the cross-product interaction term of number of mother alcohol dependence symptoms multiplied by number of developmental periods of maltreatment exposure. Subsequently, a second structural model was tested such that childhood externalizing symptomatology, internalizing symptomatology, and peer acceptance were specified as mediators of the relation between predictors and the latent outcome of young adult problematic alcohol use. The residual correlations between the mediators were estimated. Mothers’ education level, assessed as a binary variable (i.e., at least high school degree versus below high school degree), and offspring sex (i.e., male, female) were additionally examined as a covariate of each mediator and outcome given empirical support for their associations with offspring developmental risk. Specifically, lower parent education level has been identified as a correlate of offspring alcohol use (e.g., Kelly et al., 2012). An individual’s sex has been identified as a predictor of symptomatology, with males more likely to exhibit externalizing behaviors (Mayes et al., 2020) and females more likely to exhibit anxiety symptoms in childhood (e.g., Altemus et al., 2014). Offspring race was initially examined as an additional covariate, but given lack of unique significant associations with mediators and outcome, was excluded from the final model.

Missing data for endogenous variables were estimated as a function of exogenous variables based on the missing at random assumption (Schafer & Graham, 2002). The weighted least square mean and variance adjusted (WLSMV) estimation was used and missing data for endogenous variables was handled using full information maximum likelihood (FIML). Model fit was evaluated using the comparative fit index (CFI), root mean square error of approximation (RMSEA), and the weighted root mean square residual (WRMR; Hu & Bentler, 1999). Mediation was tested using 95% asymmetric confidence intervals (CIs) through RMediation (Tofighi & MacKinnon, 2011). Confidence intervals that did not include the value of zero determined significant mediation.

## Results

### Preliminary data analyses

Zero-order correlations for all measures can be seen in Table 1. As anticipated, the number of developmental periods in which maltreatment occurred was associated with greater childhood conduct problems (*r* = .18, *p* < .01), greater childhood depressive symptomatology (*r* = .15, *p* < .01), and greater likelihood of affiliation with substance-using peers or siblings during childhood (*r* = .14, *p* < .01). Exposure to mother alcohol dependence symptoms was associated with greater depressive symptomatology (*r* = .13, *p* < .01) and greater likelihood of affiliation with substance-using peers (*r* = .18, *p* < .01). The number of developmental periods of maltreatment and mother alcohol dependence symptoms each were associated with offspring alcohol dependence symptoms in emerging adulthood (*r* = .13, *p* < .05; *r* = .21, *p* < .01, respectively). Moreover, number of developmental periods was associated with parent alcohol dependence symptoms (*r* = .14, *p* < .01).

### Measurement model

**Figure 1.**
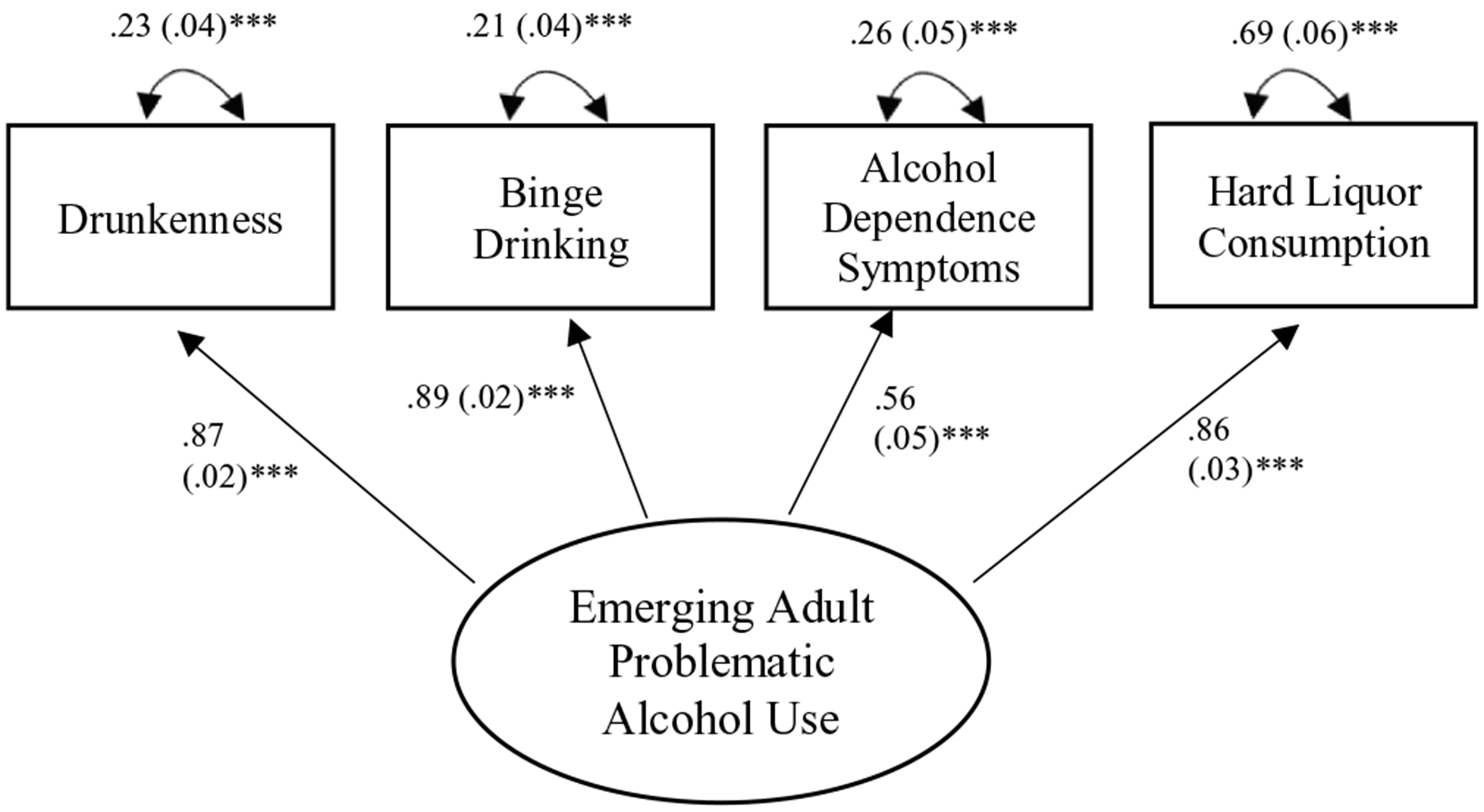
Measurement model.

Regarding the measurement model, the model evidenced good fit to the data, *χ*^2^(2) = 3.55, RMSEA = .09 (90% CI = .03–.15), CFI = .98, SRM*r* = .01. With respect to the problematic alcohol use latent factor, binge drinking, drunkenness, hard liquor consumption, and alcohol dependence symptoms each evidenced large and significant factor loadings (λ = .88, λ = .90, λ = .59, and λ = .88, respectively; *ps* < .001) (See Figure 1).

### Structural model

**Figure 2.**
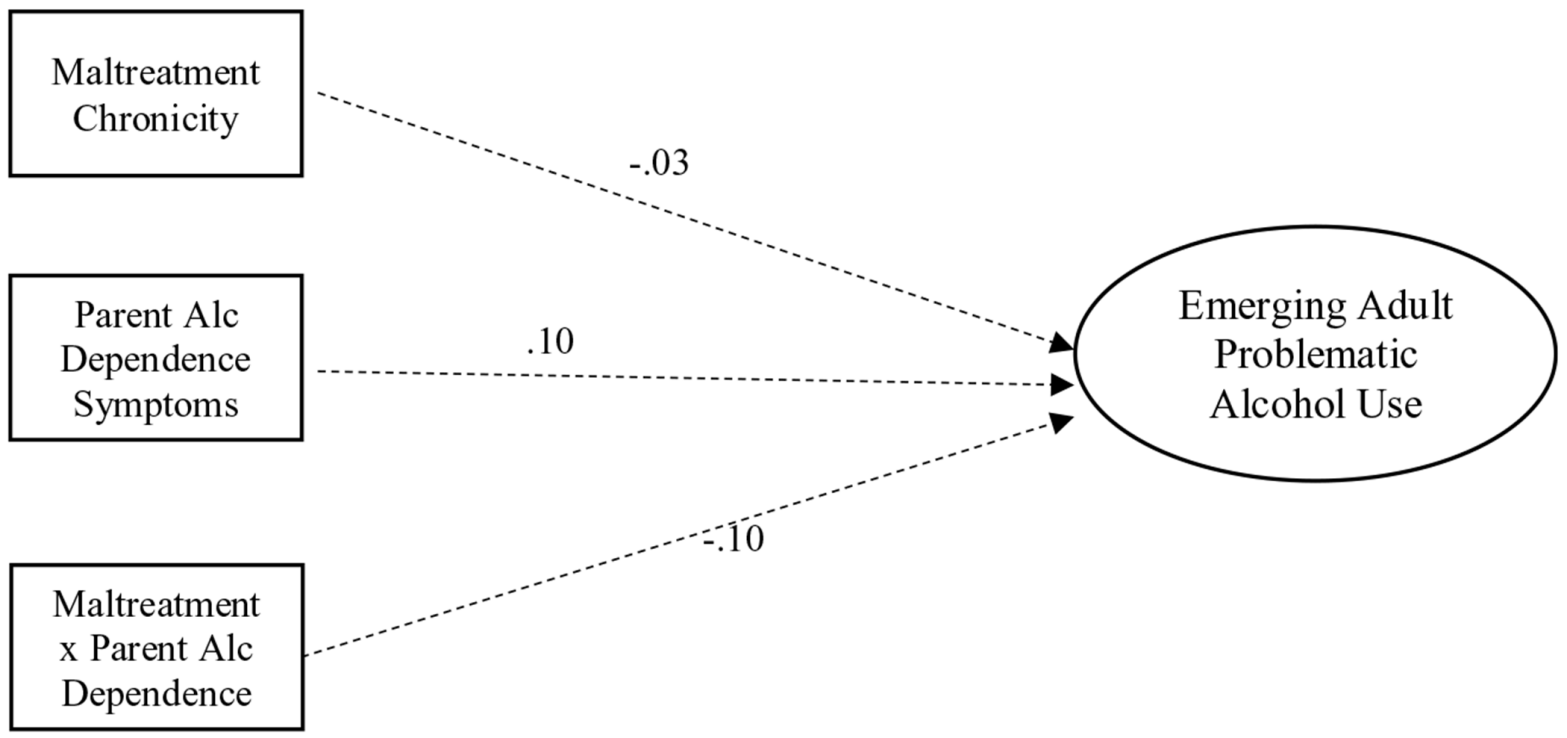
Structural model without mediators. Note. Dashed lines indicate nonsignificant paths.

The first structural model evidenced good fit to the data, *χ*^2^(11) = 37.72, RMSEA = .09 (90% CI =.06–.13), CFI = .96, SRMR = .04. Significant path coefficients are depicted in Figure 2. Maltreatment chronicity and maternal alcohol dependence were not significantly associated with emerging adulthood alcohol use (*β* = −.03, *p* = .61; *β* = .10, *p* = .15). Similarly, the interaction of maltreatment and mother alcohol dependence was not a significant predictor of emerging adult alcohol use (*β* = −.10, *p* = .12).

**Figure 3.**
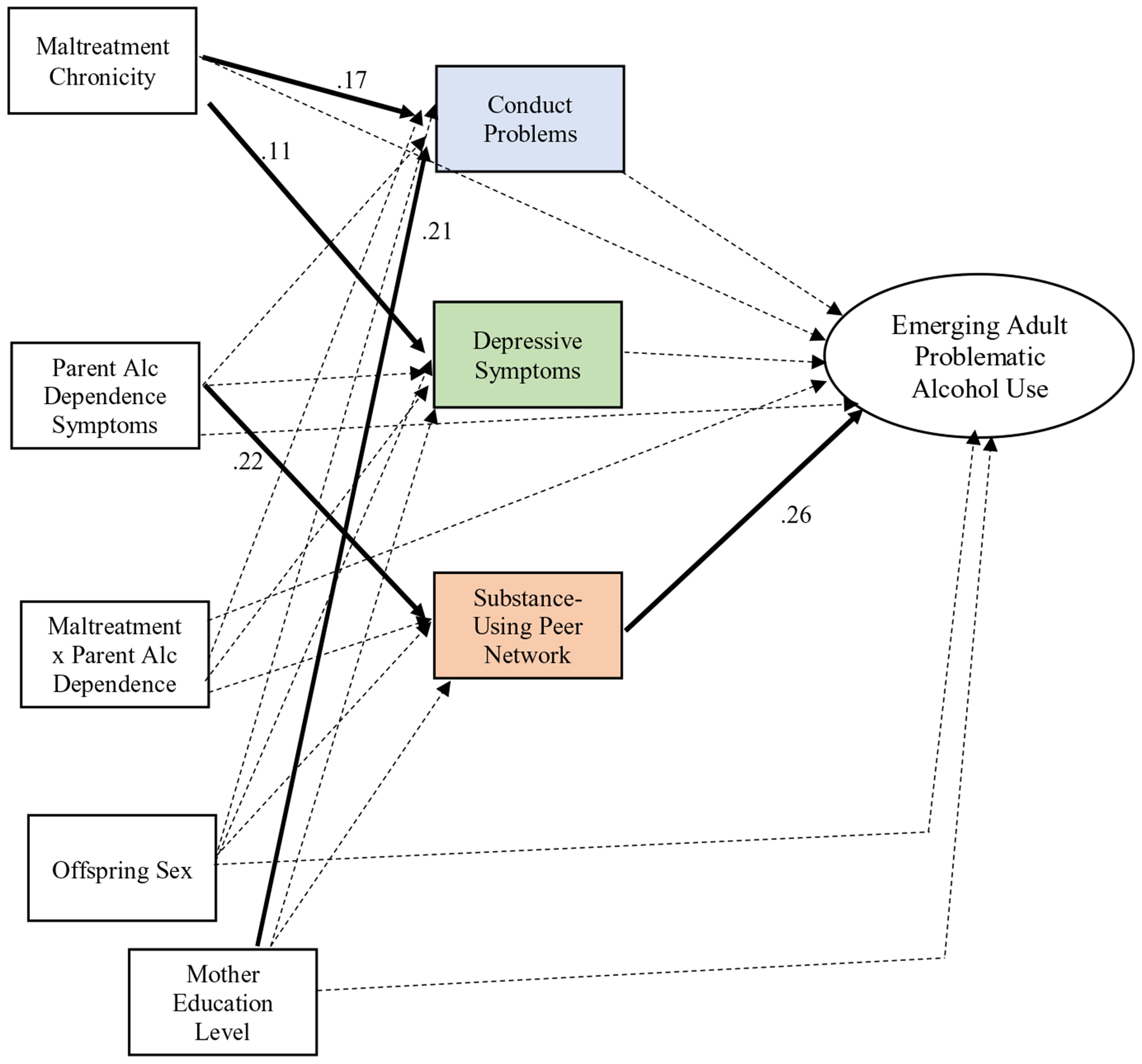
Structural model with mediators. Note. Significant paths are bolded. Nonsignificant paths are dashed. Coefficients only included for significant paths. Mother attainment of high school degree or above coded as 1, less than high school degree coded as 0; Male sex coded as 1, female sex coded as 0.

The subsequent structural model, testing mediation pathways, evidenced good fit to the data, *χ*^2^(26) = 45.58, RMSEA = .05 (90% CI = .02–.07), CFI = .94, WRMR = .04 and distinct findings from those of the first structural model. Significant path coefficients are depicted in Figure 3. Greater maltreatment chronicity significantly predicted greater childhood conduct problems (*β* = .17, *p* < .01) over and above the effect of mother alcohol dependence symptoms (*β* = .04, *p* = .58). The interaction of maltreatment and mother alcohol dependence was not a significant predictor of externalizing problems (*β* = −.06, *p* = .32). Childhood conduct problems did not predict alcohol use among emerging adult offspring (*β* = .08, *p* = .36). Greater maltreatment chronicity significantly predicted greater childhood depressive symptoms (*β* = .11, *p* < .05) over and above the effect of mother alcohol dependence symptoms (*β* = .09, *p* = .06) and the interaction of maltreatment and mother alcohol dependence (*β* = −.07, *p* = .16). Childhood depressive symptoms were not associated with emerging adult alcohol use (*β* = −.07, *p* = .30).

Additionally, greater mother alcohol dependence symptoms predicted greater likelihood of affiliation with substance-abusing peers and siblings (*β* = .22, *p* < .01), over and above the effect of maltreatment chronicity (*β* = .10, *p* = .12) and the interaction of maltreatment and mother alcohol dependence (*β* = −.01, *p* = .95). Moreover, childhood affiliation with substance-using peers and siblings predicted greater alcohol use among emerging adult offspring (*β* = .26, *p* < .01). None of the exogenous predictors (i.e., maltreatment chronicity, parent alcohol dependence symptoms, interaction of maltreatment and parent alcohol dependence) had a direct unique association with offspring emerging adult alcohol use (*β* = −.06, *p* = .32; *β* = .08, *p* = .32; *β* = −.08, *p* = .24, respectively). Regarding the mediation pathways tested, significant mediation was observed via the peer affiliation pathway, such that being reared by a mother with greater alcohol dependence symptoms predicted affiliation with peers and siblings engaging in substance use during childhood, which in turn predicted greater emerging adulthood alcohol use (see Table 2 for all indirect effects).

With respect to the covariates of interest, mothers’ education level was significantly associated with greater offspring depressive symptoms and peer affiliation (*β* = −.12, *p* < .05; *β* = −.20, *p* < .01, respectively). Mother’s education level was not associated with offspring conduct problems (*β* = −.09, *p* = .11) or offspring alcohol use as emerging adults (*β* = −.02, *p* = .73). Males were marginally significantly more likely to exhibit concerning alcohol use in emerging adulthood (*β* = .13, *p* = .07) and more likely to exhibit greater conduct problems in childhood (*β* = .21, *p* < .001). However, sex was not associated with depressive symptomatology (*β* = .02, *p* = .66) or peer affiliation (*β* = −.01, *p* = .87). Regarding residual correlations observed among mediators, there was a significant residual correlation between conduct problems and depressive symptoms (*β* = .32, *p* < .001), and between conduct problems and affiliation with substance-using peers (*β* = .46, *p* < .001). The residual correlation between peer affiliation and depressive symptoms was nonsignificant (*β* = .12, *p* = .10).

## Discussion

In the present study, we investigated the unique and amplifying effects of exposure to parental alcohol use disorder and exposure to childhood maltreatment on the development of problem drinking in emerging adulthood. We also tested whether childhood externalizing symptoms, internalizing symptoms and affiliation with a substance-using peer network underlie these associated forms of early familial adversity. There were several notable findings of the present study. First, consistent with prior research, we observed multifinality in the effects of chronic maltreatment on offspring development, such that maltreatment leads to multiple forms of maladjustment. Specifically, even in the context of exposure to mother alcohol use disorder, more chronic maltreatment was uniquely associated with greater childhood conduct problems, supported by prior research (e.g., Manly et al., 2001; Stewart et al., 2008) and greater depressive symptomatology, aligning with Ethier et al. (2004).

Our findings that maltreatment uniquely conferred risk for symptomatology can be contextualized through our understanding of the influential role of maltreatment in challenges with emotion regulation, emotional suppression (e.g., Gruhn & Compas, 2020), and dysregulated stress response system activity (e.g., McLaughlin et al., 2014). Difficulties managing emotions and disruptions to normative stress response system activity (e.g., heightened cortisol reactivity) have each been identified as predictors of symptomatology (e.g., Hagan et al., 2014; Kim & Cicchetti, 2010, respectively), with robust support for these pathways linking maltreatment exposure and behavioral challenges, including externalizing and internalizing symptomatology.

Interestingly, mother alcohol dependence was uniquely associated with greater affiliation with substance-using peers and siblings during childhood, aligning with the prior assertations that substance use among parents decreases the amount of monitoring they provide to adolescents (e.g., Chassin et al., 1996; Sher et al., 2005). Decreased parental monitoring can result in fewer restrictions imposed regarding the types of peers with whom offspring can engage and ultimate promote greater accessibility of substances and heavier use of substances among adolescents (Clark et al., 2012; Van der Vorst et al., 2006).

Mother alcohol dependence was indirectly associated with emerging adult alcohol use via the peer and sibling affiliation pathway, such that exposure to mother AUD predicted greater affiliation with substance-using peers and older siblings in childhood, which in turn predicted greater alcohol use as young adults. However, externalizing and internalizing symptomatology during childhood did not uniquely predict emerging adult alcohol use and did not mediate the effects of either form of adversity. Taken together, when examining maltreatment and mother AUD in tandem, the development of symptomatology appears to be unique to maltreatment exposure, whereas affiliation with substance-using peers and siblings appears to be unique to maternal AUD and acts as a key mechanism in the intergenerational transmission of substance use between mothers and offspring. This mechanistic role aligns with extant research supporting peer affiliation as one of the most robust predictors of substance use in adolescence and emerging adulthood (Cambron et al., 2018; Chassin et al., 2013; Lee et al., 2017). These findings fit within the social learning perspective, such that youth who are affiliated with peer groups and older siblings engaging in and normalizing substance use are more likely to model substance-using and other deviant behaviors. Moreover, engaging in substance use during preadolescence and adolescence, which are critical periods for social development, may facilitate group belongingness and acceptance by peers (e.g., Ellis & Wolfe, 2008). Despite our findings of an indirect pathway to offspring development, we observed that maltreatment and mother alcohol dependence did not uniquely directly predict offspring alcohol use in emerging adulthood.

One surprising result was that the interaction of maltreatment and mother AUD was not associated with any pathways of interest, such that neither form of adversity amplified the effect of the other when measured in tandem. This result may be due to the methodology of the current study, as we did not account for the timing of maltreatment relative to mother alcohol dependence symptoms. Specifically, mother alcohol dependence may have temporally preceded, occurred in tandem, or occurred after exposure to maltreatment. Moreover, we know that maltreatment exposure occurred during the child participants’ lifetimes, whereas mother alcohol dependence, evaluated as it pertained to a mother’s lifetime, could have transpired prior to their child’s birth. As such, amplification of these forms of adversity may have been weakened if mother AUD was resolved prior to offspring exposure.

Also counter to expectations, conduct problems did not mediate the effect of either form of adversity on offspring alcohol use. Despite prior research suggesting that externalizing behaviors (e.g., aggression) mediate the association between maltreatment and offspring substance use (e.g., Cicchetti & Handley, 2019; Oshri et al., 2011), we recognize that the experience of conduct problems is associated with engagement in deviant peer groups, as captured by the peer and sibling affiliation pathway. Our results suggest that when considering both mechanisms simultaneously, affiliation with substance-using peers during the school age years is a more salient predictor of later alcohol use problems, relative to conduct problems, during this developmental period.

The smaller effect sizes observed in our model may be a reflection of the complexities of the processes of maltreatment and parent alcohol use exposure. For example, maltreatment represents one of the more complex constructs in the social sciences (Jackson et al., 2019; Warmingham et al., 2019), making it difficult to capture all of the developmental processes informed by the experience of maltreatment. We investigated the externalizing, internalizing, and peer affiliation pathways given robust empirical support for their roles as mechanisms in the effects of various forms of early adversity (e.g., Chassin et al., 2013; Cicchetti & Handley, 2019). However, we recognize that alternate pathways may account for some of the effects of maltreatment and parent AUD, informing the effect sizes that we observe in our current model.

Potential confounders of the effects of interest include poverty, which can influence youth socioemotional development and well-being (e.g., Chaudry & Wimer, 2016). However, poverty is a variable that was handled by design of the study, given that all children were experiencing poverty to be included in the sample. We additionally acknowledge that the behavior and functioning (e.g., AUD exposure) of other caregiving figures beyond the mother (e.g., mother’s romantic partner) can be a confounding influence on offspring development. However, the majority of families involved in the research summer camp program were comprised of single-parent households with separate caregivers (e.g., fathers) who were not accessible for contact due to factors including incarceration or abandonment. As such, we did not have consistent data on the behaviors and health history of those individuals.

An additional limitation worth noting is that we used a variable-centered approach, such that the exposure to maltreatment and mother alcohol dependence are measured as discrete, co-occurring categories of adversity. However, we recognize the potential utility of using a person-centered analytic approach to identify naturally occurring constellations and heterogeneous patterns of adverse familial experiences. Despite these limitations, the results of the present study remain meaningful in elucidating unique pathways of risk for individuals exposed to both maltreatment and mother AUD, relative to prior work that captures these experiences in separate models, or as a cumulative adversity measure.

Strengths of the present study include the multi-informant nature of our analyses, including coded CPS records, child self-report, and maternal report assessments. Additionally, this is among the first studies to capture the pathways of risk from exposure to maltreatment and mother AUD simultaneously in a prospective model, given the highly co-occurring nature of these processes (e.g., Appleyard et al., 2011; Hayre et al., 2019). We evaluated effects of maltreatment chronicity, rather than subtype, given the assertions of extant research that chronicity, among other characteristics, can provide a richer level of detail regarding the maltreatment experience (e.g., Jackson et al., 2019). Moreover, this study has a large sample size and is longitudinal and prospective in design, such that we capture long-term effects of high-risk adverse experiences following approximately a decade of development.

In conclusion, the present study examined the unique effects of two highly co-occurring experiences of early adversity within the family, maltreatment and mother AUD, on offspring alcohol use in emerging adulthood. Further, we sought to examine the mechanistic roles of youth externalizing and internalizing symptomatology and affiliation with substance-using peers and siblings. We identified distinct effects of maltreatment versus mother AUD, such that maltreatment informs the development of symptomatology over and above the effect of parent AUD. Conversely, being reared by a mother with AUD uniquely predicts affiliation with substance-using peers and older siblings. As such, peer and sibling affiliation was a key mechanism in the intergenerational transmission of substance use, bolstering the findings of prior empirical work asserting the robust mechanistic role of peer affiliation within exposure to parent AUD (e.g., Chassin et al., 2013; Chuang et al., 2005; Haller et al., 2010).

With respect to clinical implications of this study, our findings underscore the reality that we should not be combining adversities together (e.g., adverse childhood experiences [ACEs] model; Felitti et al., 1998). Instead, we should be considering the unique effects of different types of adversity, such that children exposed to maltreatment and children exposed to parental AUD require different mitigation strategies, with children enduring maltreatment receiving interventions focused on symptomatology, whereas children of parental AUD should receive intervention targeting peer group affiliation. In sum, the results of the current study indicate that simultaneous examination of maltreatment and mother AUD is a worthy avenue for increasing our understanding of the complex processes through which childhood adversity confers risk for youth and emerging adults.

## Figures and Tables

**Table 1.** Zero-order correlations among study variables

	1.	2.	3.	4.	5.	6.	7.	8.	9.	10.
1. Number of Mal Dev Periods										
2. Parent Alc Dependence	.14**									
3. W1 Conduct Problems	.18**	.05								
4. W1 Depressive Symptoms	.15**	.13**	.24**							
5. W1 Peer/Sibling Affiliation	.14***	.18***	.37***	.18**						
6. Mother Education Level	−.16**	−.002	−.07	−.12**	−.12**					
7. Offspring Sex	−.002	.05	.19**	.04	.01	−.01				
8. Offspring Drunkenness	.04	.02	.12*	.03	.12*	−.03	.01			
9. Offspring Binge Drinking	.03	.03	.19**	.07	.17**	−.03	.14**	.77**		
10. Offspring Alc Dependence	.13*	.21**	.13*	.09	.21***	−.07	.05	.53**	.48**	
11. Offspring Hard Liquor Consumption	.01	.06	.18**	.007	.16**	.002	.16**	.75**	.77**	.48**

* *p* < .05,

** *p* < .01,

*** *p* < .001.

W1 represents measurement during the wave 1 assessment period.

**Table 2.** Indirect effects

Indirect pathways	β	95% CI
Maltreatment → Externalizing → Offspring Alcohol Use	.016	−.018 to .058
Parent AUD → Externalizing → Offspring Alcohol Use	.002	−.009 to .019
Maltreatment & Parent AUD → Externalizing → Offspring Alcohol Use	−.003	−.019 to .006
Maltreatment → Internalizing → Offspring Alcohol Use	−.009	−.034 to .009
Parent AUD → Internalizing → Offspring Alcohol Use	−.004	−.018 to .005
Maltreatment & Parent AUD → Internalizing → Offspring Alcohol Use	.002	−.004 to .015
Maltreatment → Peer/Sibling Affiliation → Offspring Alcohol Use	−.034	−.043 to .004
Parent AUD → Peer/Sibling Affiliation → Offspring Alcohol Use	**.050**	**.008 to .097**
Maltreatment & Parent AUD → Peer/Sibling Affiliation → Offspring Alcohol Use	−.001	−.030 to .027

*Note*. Bolded values indicate statistical significance, α = .05.
